# Clinical Hypnosis, an Effective Mind–Body Modality for Adolescents with Behavioral and Physical Complaints

**DOI:** 10.3390/children4040019

**Published:** 2017-03-24

**Authors:** Anju Sawni, Cora Collette Breuner

**Affiliations:** 1 Department of Pediatrics, Hurley Children’s Hospital/Hurley Medical Center, Michigan State University College of Human Medicine, Flint, MI 48503, USA; 2 Department of Pediatrics, Adolescent Medicine Division and Department of Orthopedics and Sports Medicine, Seattle Children’s Hospital, University of Washington, Seattle, WA 98105, USA; Cora.Breuner@seattlechildrens.org

**Keywords:** mind–body therapies, adolescents, pain, headache, stress, anxiety, depression, hypnosis, guided imagery, mindfulness, emotional regulation

## Abstract

Mind–body medicine is a system of health practices that includes meditation/relaxation training, guided imagery, hypnosis, biofeedback, yoga, art/music therapy, prayer, t’ai chi, and psychological therapies such as cognitive behavioral therapy. Clinical hypnosis is an important mind–body tool that serves as an adjunct to conventional medical care for the adolescent patient. Clinical hypnosis specifically uses self-directed therapeutic suggestions to cultivate the imagination and facilitate the mind–body connection, leading to positive emotional and physical well-being. There are many similarities between clinical hypnosis and other mind–body/self-regulatory modalities such as visual imagery, mindfulness meditation, yoga, and biofeedback that incorporate experiential learning and mechanisms for change. They may be viewed as subtypes of the hypnotic experience and share the common experience of trance as the entrée into self-empowered change in physiologic and psychological states. Clinical hypnosis can be used by health care providers to teach adolescents coping skills to deal with a wide variety of conditions such as chronic headaches, recurrent abdominal pain, anxiety, depression, grief and bereavement, phobias, anger, family stressors, sleep disorders, or enuresis. Clinical vignettes are given to help illustrate the effectiveness of hypnosis in adolescents.

## 1. Introduction 

Mind–body medicine is a philosophy and a system of health practices that enlist the mind in improving emotional well-being and physical health. These practices include meditation/relaxation training, guided imagery, hypnosis, biofeedback, yoga, art/music therapy, prayer, t’ai chi, and psychological therapies such as cognitive behavioral therapy.

Mind–body therapies focus on balancing the autonomic nervous system (ANS) by activating the parasympathetic branch of the ANS to reduce the sympathetic physiological response to stress and by regulating the hypothalamic pituitary adrenal axis, both of which are indicators of stress. This reduction of stress on the mind and body may help control or reverse certain underlying disease processes. Evidence suggests that the mind and body have constant bidirectional communication through neuro-endocrine, neuro-chemical, immunological, and energetic pathways.

Higher cognitive centers and limbic emotional centers are capable of regulating virtually all aspects of the immune system and therefore have a profound effect on health and illness-termed psychoneuroimmunology [1,2,3].

These therapies may improve the quality of life and reduce the physical symptoms for adolescents with various chronic diseases.

Data from the 2012 National Health Interview Survey show that mind–body therapies were used in adolescents aged 13 to 17 years, and more often among females versus males (5.7% vs. 1.7%). Children and youth were more likely to use mind–body therapies for pain-related conditions or emotional, behavioral, or mental conditions and if they received specialty or mental health care. The most common reasons for the use of mind–body therapies were to improve overall health/general wellness, to reduce stress levels or relax, and to feel better emotionally [4,5].

Mind–body therapies such as clinical hypnosis are considered safe and can be effective for adolescents with medical conditions such as tension or migraine headaches, enuresis, recurrent abdominal pain, constipation, sleep difficulties, acute or chronic pain, anxiety, and other emotional and stress-related symptoms [6,7].

## 2. Stress and the Developing Adolescent Brain

Adolescence is a period of rapid physical growth, intellectual and cognitive development, as well as a time for creating autonomy, and promoting both self-esteem and strong peer relationships. During adolescence, teens experience the ebb and flow of emotions between self-confidence, insecurity, invincibility, anxiety, worry, doubt, and self-worth as a result of rapid changes occurring in the brain and neuro-biological and neuro-endocrine systems.

The rapidly developing adolescent brain is sensitive to stress and adverse events due to changes in hormones and the plasticity in the structure and function of the brain [8].

Brain development during adolescence involves changes in the frontal and parietal cortices, the site of higher-order cognitive and socioemotional processes (i.e., executive function). The cortex is fine-tuned through synaptic pruning in areas that play a role in judgment, impulse control, planning, and emotion regulation [9,10,11].

Glucocorticoid receptors are found in the amygdala, hippocampus, and prefrontal cortex (PFC); exposure to stressful experiences (leading to increase in glucocorticoids) has been shown to alter the size and neuronal architecture of these areas as well as lead to functional differences in learning, memory, and other aspects of executive functioning. Specifically, chronic stress is associated with hypertrophy and over-activity in the amygdala and orbitofrontal cortex, and can lead to loss of neurons and neural connections in the hippocampus and medial PFC. The PFC turns off the cortisol response and regulates the autonomic balance (i.e., sympathetic versus parasympathetic effects), it also plays an important role in the development of executive functions, such as decision-making, working memory, behavioral self-regulation, and mood and impulse control [12]. The consequences of structural changes in the brain include more anxiety related to both hyper activation of the amygdala and less control as a result of PFC atrophy, as well as impaired memory and mood control due to hippocampal reduction [13].

The adolescent brain is particularly vulnerable to stressors in the youth’s social and emotional environment. Executive functions, such as the ability to direct attention and solve problems efficiently, the awareness of threat, and effective fear processing are diminished when there are significant emotional stimuli during adolescence [14,15,16,17]. Rapid neurobiological changes during times of emotional stress may predispose adolescents to difficulties with emotion regulation [8]. Difficulties in emotion regulation are a feature of many emotional and behavioral problems seen in adolescents, including anxiety, depression, conduct problems, cutting, disordered eating, and substance abuse [18,19,20].

The prevalence of mental health disorders is 22% in adolescents 13 to 18 years old. Anxiety disorders are the most common condition (31.9%), followed by behavior disorders (19.1%), mood disorders (14.3%), and substance use disorders (11.4%). Approximately 40% of those with one class of disorder also meet criteria for another class of lifetime disorder [21,22].

With increasing exposure to technology and screen time (on smart phones, computers, television, and social media sites), more mental health concerns, and persistent economic, racial, and ethnic disparities in health status, we now have new morbidities in adolescents called the ‘millennial morbidities’. These morbidities may lead to stress-related problems such as chronic headaches, abdominal pain, anxiety, depression, and other emotional problems [23].

Adolescents who experience emotional stress often complain of chronic physical symptoms that respond poorly to standard medications. Cognitive and emotion regulation, including the ability to modulate responses to stress, is increasingly found to contribute to overall adjustment, including social emotional development (e.g., peer relations). Mind–body/self-regulation therapies such as clinical hypnosis operate at the physiological, attentional, emotional, cognitive, and behavioral levels and teach adolescents’ coping skills to control their inner wellbeing with respects to thoughts, emotions, attention, and performance [24,25]. Research shows that mind–body/self-regulation therapies can help develop appropriate connections between the relevant prefrontal structures in adolescents, thereby stabilizing arousal and reducing harmful risk-taking behaviors [26]. They may reduce the impact of stress-related conditions, lessen depression and anxiety, alleviate pain, improve quality of life, and increase emotion regulation and subjective well-being [27,28,29,30,31,32,33,34].

## 3. Clinical Hypnosis: An Effective Mind–body Modality for Adolescents 

There are many similarities between clinical hypnosis and other mind–body/self-regulatory modalities such as visual imagery, mindfulness mediation, yoga, and biofeedback that incorporate experiential learning and mechanisms for change. They all have some overlap and may be viewed as subtypes of the hypnotic experience and share the common experience of trance (what happens when we engage in changing our mind) as the entrée into self-empowered change in physiological and psychological states. However, clinical hypnosis specifically uses self-directed therapeutic suggestions to cultivate the imagination and facilitate the mind–body connection, leading to positive physical, emotional, and behavioral change. Using brain imaging, cognitive neuroscientists have identified the power that therapeutic suggestions have on attention functions and associated brain networks and their impact on physical and mental experience [35,29].

Clinical hypnosis is a teachable coping skill that most adolescents (except those with moderate to severe mental retardation) are able to learn with minor effort, and it is safe, effective, and has no adverse side effects in trained hands.

Although there is no universally agreed-upon definition of hypnosis, it can be best described as the cultivation of the imagination in an altered state of consciousness (awareness and alertness) within a focused state (with or without physical relaxation), in which an individual is selectively focused, absorbed, and concentrating upon a particular idea or image aimed at improving mental or physical health. In adolescents, visual imagery/progressive muscle relaxation is often used to get into this altered state of awareness. It is an internal, imaginative process (similar in feeling to daydreaming and/or using the imagination to see, hear, touch, smell, and taste things) that is used to focus attention and become absorbed so that a hypnotic state/trance is entered into. In this state, perceptions and sensations can be enhanced, modified, or changed to inhibit and control reflexive actions, delay gratification, use problem-solving strategies, increase self-esteem, and decrease anxiety, stress, or discomfort. Hypnosis is a natural state and most adolescents move in and out of spontaneous hypnotic-like states as they focus their concentration, for example, while playing video games, “text-messaging”, watching favorite movies, listening to music/stories, or otherwise engaging in fantasy/daydreaming [7].

Hypnosis in adolescents is more permissive and less directive than in adults, as it utilizes the natural hypnotic abilities that teens bring to the clinical encounter. Adolescents can enter into the hypnotic state easily and rapidly and, while in this state of deep concentration, are highly responsive to therapeutic suggestions/goals (“hypnotherapy”). Examples of therapeutic suggestions include decreasing or eliminating undesirable symptoms, reframing and rethinking distorted thoughts about situations and stressors, building positive expectations, re-enforcing control over reaction to problems/situations, and strengthening the concept/belief in the ability of the mind and body to work together to create desirable changes in behavior/outcome. Hypnotherapy allows the adolescent to gain a sense of control, increase self-esteem and competence, and reduce stress.

Clinical hypnosis is indicated when (1) an adolescent is responsive to hypnotic suggestions; (2) a problem is treatable with hypnosis; (3) good rapport exists between the adolescent and provider; (4) the adolescent is motivated to remedy the problem; and (5) no iatrogenic harm is anticipated by use. Caution is indicated in adolescents with a history of physical, sexual, or emotional abuse and those with post-traumatic stress disorder (PTSD), in which case coordination of care with a qualified mental health expert is strongly advisable.

Clinical hypnosis is an effective and powerful tool and with appropriate clinical pediatric hypnosis training (skills-based pediatric hypnosis workshops, www.nphti.org). Pediatric/adolescent health care providers’ can help adolescents cope and deal with a wide variety of conditions such as chronic headaches/recurrent abdominal pain, anxiety, depression, grief and bereavement, phobias, anger, family stressors, sleep disorders, or enuresis [36,37,38,39,40,41,42,43,44,45,46,47].

Hypnosis can also be effective for managing procedure pain [48] and chronic pain related to disease—i.e., malignancy, sickle cell, chronic headaches, and abdominal pain (recurrent abdominal pain, inflammatory bowel disease) [49,50,51,52,53,54].

Clinical hypnosis/self-regulation therapies also can be helpful when integrated into a multimodal treatment plan for adolescents with Attention deficit hyperactivity disorder (ADHD). Hypnosis can be beneficial in teaching them to quiet their mind, slowing and focusing, so that they can sort out frustration or anger produced when the mind works faster than the ability to communicate, and direct them in a positive manner [55,56,57,58].

For more detailed descriptions and references of the wide range of clinical conditions for which hypnosis is applicable and effective in adolescents, readers can consult the two standard textbooks on clinical hypnosis by Kohen and Olness, Sugarman and Wester [59,60], as well as an excellent review article by Kohen and Kaiser [61].

## 4. Application of Clinical Hypnosis—Clinical Vignettes

Through clinical vignettes, this article illustrates common applications of clinical hypnosis in adolescent health care. These vignettes demonstrate the ease, value, and benefit of having pediatric/adolescent health care providers empower and teach clinical hypnosis strategies to adolescents.

The success of clinical hypnosis depends on establishing a strong rapport with the adolescent and individualizing the therapy to the specific goals and characteristics of the adolescent. During the initial visit, the clinician learns the adolescent‘s personal and family history, and assesses motivation, expectations, strengths, and other internal resources—such as capacity to self-regulate their emotions—the clinician should emphasize that the adolescent is in control. They should offer choices and options and teach the adolescent that they can use the skill (clinical hypnosis) when he/she chooses. The clinician should offer to be the teacher or coach, emphasize the adolescent’s ability for control and mastery, and utilize the teen’s language and imagery. They should reinforce whatever happens, get feedback from the teen, and address anxiety as well as pain. It is important to have a follow-up plan, such as emphasizing the importance of clinical-hypnosis practice and keeping a calendar of the practice and symptoms (this can easily be done on the teen’s smart-phone; the majority of teens have a one). The provider should schedule a follow up appointment in a few weeks to reassess/reinforce progress. The use of other cognitive and behavioral strategies should be incorporated as well to address the symptom.

### 4.1. Clinical Vignette 1: Chronic Daily Headaches

A 16-year-old female was referred by her neurologist for chronic headaches. Her neurological work up for headaches was negative, with no anatomical or pathological etiology. She had a history of daily headaches, that did not wake her up from sleep but did cause her to miss school and not socialize with her friends. She took over-the-counter ibuprofen/acetaminophen with little relief. She would sleep to help her headaches.

Past medical history and social history were non-contributory. No history of depression, anxiety, or school problems. Family/home life was stable with no acute/chronic toxic stressors.

Mental status evaluation revealed normal cognition and no apparent psychiatric disorder such as a conversion disorder, psychosis, or thought disorder.

The initial session with her and her parents focused on obtaining a thorough history, performing a physical exam, and reviewing labs/imagery. Pain assessment scale using range of 0–10 was used (10 = worst imaginable headache, 0 = no headache, her average score was 8). When asked at which number she would be able to go to school/function so that the headache did not bother her, she replied ≤3.

The clinician then educated the patient and parents on the mind–body connection, with a more formalized introduction/discussion and demystification of hypnosis, emphasizing that learning strategies such as clinical hypnosis can be very effective in managing headache pain.

It was explained that clinical hypnosis could help her take control of her pain/headaches and she could do something so that her headaches would not bother her anymore. The patient and parents were receptive to learning clinical hypnosis. The patient was motivated to get rid of her headaches and resume her normal life.

A second visit was scheduled to teach the patient clinical hypnosis techniques. When designing a hypnotic or hypnosis/trance experience, the clinician utilizes the adolescent’s imagination and perception of the symptom and how it affects them as well as probes into the adolescent’s interests, strengths, and goals. The adolescent is taught to use techniques such as progressive muscle relaxation, focused breathing, and visual imagery to help them get into a relaxed and focused state (with or without physical relaxation). In this relaxed state, the brain is more focused and receptive to learning techniques to feel better and ultimately make the headache pain dissipate. Prior to incorporating visual imagery and giving a therapeutic suggestion to alleviate the symptom or problem, the clinician asks the adolescent what they would like to imagine that is fun and makes them happy (i.e., a favorite place or favorite things) and if they would like to imagine doing that to help them get into a deeper relaxed state (this reinforces that the teen is in control and empowers them). In this deeper relaxed state, the mind is open and receptive to ideas to help them with their symptoms. In this case the clinician asked the patient to draw a picture of what her headache felt like to her. She drew a picture of a hammer pounding her head. Then both the clinician and the patient devised a method to stop the hammer from pounding her head. The patient used her imagination to visualize having a stop sign come up before the hammer hit her head which would stop the hammer and thus would stop the headache. She then practiced visualizing the stop sign coming up and saying stop to the hammer before it hit her head (the therapeutic suggestion). The clinician then developed a concrete plan with the teen to help practice her clinical hypnosis techniques. Some clinicians ask their patients to keep a calendar of their practice (easy to do on their smartphone). The focus is on practicing clinical hypnosis, not on the symptoms. At the follow up visit a month later, she was practicing her clinical hypnosis, she had kept a calendar of when she practiced hypnosis and pain scale of the headaches. Her headaches were infrequent and not interfering with her daily activities or school.

### 4.2. Clinical Vignette 2: Needle Phobia

This was a 14-year-old male was referred by his primary care doctor to learn clinical hypnosis for needle phobia. He became very anxious and hysterical prior to obtaining any immunizations or blood draws and was therefore behind on immunizations and screening lab tests.

Past medical history was significant for attention deficit hyperactivity disorder, treated with methylphenidate. There was no history of other psychiatric diagnosis.

He was motivated to learn clinical hypnosis. He recognized that he needed immunizations to stay healthy but was afraid of shots. He was motivated and willing to overcome his “fear of shots”. The clinician discussed mind–body/clinical hypnosis techniques with him and used a distraction technique (listening to his music on his iPod) and the hypnotic concept of disassociation i.e., dissociate body and mind, to encourage him to give his arm to the nurse to let her give him the shot so that he could stay healthy, and while she did what she needed to do, he could focus on something else like listening to his music. Also, exposure therapy with desensitization is the treatment of choice for phobias. He was exposed to the equipment (e.g., cleaning swab, syringe, needle) and procedure for injections, so those items no longer created panic symptoms. A “pretend needle” was used to do practice session. After rehearsing this for one session he then “practiced getting the shot” (exposure therapy), while listening to his music (distraction), “gave his arm to the nurse” (disassociation). He did well in the practice session. The patient came back two days later and went through the clinical hypnosis technique of distraction and disassociation and successfully received his immunizations.

### 4.3. Clinical Vignette #3: Primary Nocturnal Enuresis

A 14-year-old male was referred by his pediatrician for management of primary nocturnal enuresis. History revealed that he had nocturnal enuresis six/seven nights/week and never had a period of complete dryness. He was in the ninth grade and played hockey with practices late at night and quite a few games away from home. He was very embarrassed about his bedwetting and thus very motivated to stop.

Family history was positive for nocturnal enuresis in father until age 16.

He reported drinking a lot of fluids after each hockey practice and game. He had a difficult time unwinding and falling asleep and was a deep sleeper.

He denied drugs, alcohol, or tobacco use and did well in school. There were no other stressors at home or school.

He was motivated to learn clinical hypnosis and stop bedwetting. The clinician discussed the mind–body connection—i.e., the brain and bladder were communicating with each other. He was told that his brain and bladder talked to each other very well, and cooperated and worked well because during the day he never wet his pants even if he had to go really badly, he could hold his pee. He was shown a drawing of genitourinary anatomy and physiology, and was engaged in building curiosity about how the body works, thus helping him be in a spontaneous hypnotic state. The drawing showed how urine was sent from the kidneys to the bladder. The bladder was shown as a container with a muscle at the tip that has a gate that is closed and keeps the urine in the bladder until he goes to the bathroom and the mother computer (i.e., the brain) gives a signal to open the gate when it is full so he can pee. The drawing showed the brain and bladder connection, with bi-directional arrows communicating between the full bladder and the brain, keeping the gate closed until he gets the message from the brain to open it.

The clinician explained, that because he was a deep sleeper, his brain and bladder did not talk well/forgot at night so he had to remind them to talk to each other like they did during the day before he went to sleep.

Then the clinician had him imagine having this conversation in his head to practice how he would have the brain and bladder talk to each other. For example, the clinician coached the patient to say “bladder, tonight let the brain know when you are full. Brain, you have a choice. When the bladder says its full, tell me to wake up, walk to the bathroom, open the gate, pee in the toilet, close the gate, and go back to my comfortable dry bed, or, instead tell the bladder gate to stay closed and locked all night until morning, when I get up from my nice, warm, dry bed and go to the bathroom”.

With either choice, the bed is dry in the morning. The patient then wanted to sleep through the night and decided to send a message to brain to keep the gate closed to his bladder till he woke up in the morning. Other behavior modification techniques such as no fluids 1 hour before bed, and bladder Kegel strengthening exercises during the day were taught. The patient kept a calendar of his clinical hypnosis practice and of dry nights.

He focused on dry nights, positive behaviors, feeling proud and good when his bed was dry, which gave him positive messages of success. He went through two sessions and at a two-month follow up his enuresis had resolved completely.

### 4.4. Clinical Vignette 4: Anxiety with Sleep Disturbance

A 13-year-old female was referred by her pediatrician for concerns regarding palpitations/panic attacks and sleep problems that started a few months earlier. The patient reported symptoms of shortness of breath, sharp chest pain, and hyperventilation. Extensive medical evaluation by a pediatrician and a cardiologist were negative for physical/anatomical abnormalities.

Patient continued experiencing palpitations and felt as if she “could not catch her breath, and her throat was closing”.

She also reported having trouble sleeping, and frequently watched scary movies on her iPad at night to help her sleep. She occasionally took melatonin to help her sleep.

Her mom reported that she was a “worrier” thought often about her safety, as well as her parents’ safety, and was concerned that something was wrong with her.

There were no recent illness/stressors in the family and no acute life changes. Family history was positive for anxiety in the father. She lived at home with parents, was an only child and got along well with both parents. She was in the eighth grade, doing virtual home schooling for past two years and was doing well. She did not like crowds and school, so parents decided to do home/virtual school. She had one close friend and cousins with whom she socialized. She liked to play the piano and do arts and crafts. She was referred to a psychologist and also saw a psychiatrist once. She was diagnosed with “social anxiety” by psychiatrist after one visit and told to take sertraline but she never started as her mother did not want to give her medications.

The clinician educated the patient and mom on the benefits of learning mind–body techniques such as focused breathing/visual imagery/clinical hypnosis, to help her cope with her panic attacks/anxiety/worried thoughts.

The patient was open and motivated to learning clinical hypnosis/relaxation techniques. She was taught focused breathing, progressive muscle relaxation, and visual imagery techniques to help her relax and get into a state of focused concentration. The clinician discussed imagining something that made her happy or feel good; her favorite imagery was being up at a lake on the boat. While in this relaxed state the clinician gave her therapeutic suggestions to notice that her breathing was calm, strong, and comfortable; her heart was beating normally and was strong and well; and she was healthy. Her parents were fine and she did not have to worry about her health or her parents’ health.

The clinician also discussed other behavioral modification techniques when she felt anxious such as coloring and journaling to help her worry less and distract her when her worry thoughts were taking over her mind. She was shown smart phone e apps (e.g., calm, breathe & relax, mind shift, stop, breathe & think) that helped her practice her breathing.

The patient was told to schedule regular practice time and instructed to keep a calendar of her clinical hypnosis exercise breathing practice and to note the days she felt good/less worried. It was recommended that she continue therapy with a psychologist. She was also advised on proper sleep hygiene techniques (i.e., no screen time one hour before bed, no TV). At a one-month follow-up she was doing better. She was practicing her clinical hypnosis/breathing/visual imagery exercises before bed, two to three times a week. Her mom noticed her overall mood was better and she had fewer panic attacks although she still got anxious, especially when she had to go to new places or places with crowds. She practiced her focused breathing when she would get anxious and she reported sleeping better. At the two month follow up, she had no more panic attacks or excessive worrying.

### 4.5. Clinical Vignette 5: Substance Abuse

A 17-year-old male was referred by his primary care doctor for management of substance abuse, anxiety, and panic attacks. The patient experienced his first acute panic attack one week prior to his visit which resulted in an emergency room (ED) visit. He was started on short acting alprazolam for acute panic attacks in ED. Was prescribed escitalopram but did not take it because he “(did not) want to take medication”. The patient had a history of smoking marijuana (MJ) daily for past four months and weekly for the past year and more recently admitted to smoking a more potent form of MJ oil called “wax/butane hash oil”. He denied any other illicit drug use or any prescription drug abuse except for an occasional beer. 

He had a past history of mild depressive symptoms/anxiety which was progressively getting worse the past year. For the past several months, he was more socially isolated and stayed in his room and smoked marijuana. He denied self-harm or suicidal thoughts.

He had a lot of worry/concerns/anxiety about his physical health especially the negative effects of marijuana on his health.

He had a lot of psychosomatic complaints, chest pain, numbness and tingling in hands/arms, nonspecific abdominal pain, back pain, and musculoskeletal pain. Since the acute panic episode, he was motivated to stop marijuana and any illicit drug use. He recognized that when he smoked the more potent marijuana “wax”, he would be more anxious, feel panicky, heart beating fast, chest hurting, nausea, abdominal pain, and numbness.

Past medical history was significant for attention deficit disorder for which he was prescribed methylphenidate for a few months but stopped two years prior. He currently reported having difficulty focusing at school and declining grades. Family history was positive for anxiety in sister.

He was a senior in high school. His hobbies included making music on his computer and playing in a band. He admitted to peer pressure to smoke marijuana. He denied being sexually active. He worked part time at a fast food restaurant.

A complete physical and neurological exam was normal. Vital signs were all normal. 

He needed a lot of reassurance that physically he was fine.

He was open to learning clinical hypnosis/focused breathing/to help with panic attacks/anxiety. He was taught progressive muscle relaxation/focused breathing/visual imagery to help him get into a relaxed alter state of focused consciousness. He visualized himself playing his electronic music/making music. While in this relaxed state, he was given therapeutic suggestions of noting that his body was healthy, that his heart, lungs, hands, and chest were all fine.

He was told he did not need drugs to make good music. He was reminded that when he smoked marijuana he did not feel good. Smoking MJ made him anxious and it was not good for his health, it made his heart beat fast, chest hurt, and nauseous. The clinician discussed the importance of regular practice of clinical hypnosis/breathing exercises. At three week follow up, he was practicing his clinical hypnosis daily and had no further panic attacks or ED visits.

He was followed monthly and for several months he continued to be pre-occupied about his physical health and have vague physical complaints. At each visit he was reassured that physically he was healthy. 

He did admit to occasionally smoking marijuana on weekends with his friends but only a few hits as he noted that when he smoked too much he would feel his heart beat fast and get anxious. He was also started on Concerta for his ADHD.

### 4.6. Clinical Vignette 6: Crohn’s Disease with Abdominal Pain

A 17-year-old female with Crohn’s disease was referred by her gastroenterologist with severe abdominal pain after multiple hospitalizations for relapse. It was hoped that clinical hypnosis would help her as she was going away to college in the fall and prior to going away was scheduled for small bowel resection/with colostomy.

Mom and patient were interested in “holistic therapies” to help her with the surgery as well as with management of her abdominal discomfort/Crohn’s disease while away at college. Mom took herbs/nutritional supplements. Clinician discussed nutrition/healthy eating with patient and mother and added probiotics and omega-3 essential fatty acid supplements to her daily medication regimen.

Patient was very motivated to feel better and manage her Crohn’s disease. The clinician taught her clinical hypnosis prior to surgery using progressive muscle relaxation/visual imagery (going to her favorite place in her imagination), then gave her therapeutic suggestions to notice how well the surgery went and how well she was healing, how healthy her gut was, and she could eat healthy foods.

She chose the suggestion “that she wanted to send a message (via text messaging) from her brain to her gut that it was healing well. The clinician reinforced that if her stomach bothered her she could breathe and go to her favorite place and imagine herself feeling good. The surgery was successful and she recovered without complications and was having less abdominal pain. She left for college feeling more confident that she could manage her distress/pain and Crohn’s disease.

The above case studies are an example of the benefits of using clinical hypnosis to help adolescents manage common medical conditions. However, it is important to keep in mind that not every adolescent will respond so quickly and positively and that proper certification is essential when applying pediatric hypnosis. Certified pediatric hypnosis training is provided by National Pediatric Hypnosis Training Institute-Inaugural Annual Skill Development Workshops in Pediatric Clinical Hypnosis (Introductory, Intermediate, and Advanced; www.nphti.org).

Not only is a trained clinical provider important for success but so is building a positive rapport with the teen and family with ongoing re-assessment, flexibility, and brainstorming for problem resolution, good outcomes, and healing. As with any skill, regular practice of clinical hypnosis by the teen enhances competence, confidence, and positive outcomes, whereas absence of regular practice is more likely to result in slower and/or less positive results. Homework assignments for the adolescent and their parents involves practice of clinical hypnosis—i.e., keeping a calendar of practice and specifying when they will practice—counseling parents about their level of involvement, and encouraging/motivating adolescents to take charge and control of their symptoms and life in order to have a positive outcome.

## 5. Conclusions 

Clinical hypnosis is a natural state that can be cultivated, with permission from the adolescent and builds on the adolescent’s existing strengths and interests. It is an altered state of consciousness (awareness and alertness) within a focused state (with or without physical relaxation), in which an individual is selectively focused, absorbed, and concentrating upon a particular idea or image aimed at improving mental or physical health. Clinical hypnosis is a teachable skill that most adolescent patients are able to learn with minor effort, and is safe, effective, and free of adverse side effects in trained hands. Hypnosis promotes the cultivation of imagination and patients’ positive expectations and motivation for success.

Hypnosis in adolescents is more permissive and less directive than in adults, as it utilizes the natural hypnotic abilities that teens bring to the clinical encounter. As a result, adolescents can enter into the hypnotic state quicker and easier than adults and are highly responsive to therapeutic suggestions/goals (hypnotherapy).

Examples of therapeutic suggestions include decreasing or eliminating undesirable symptoms, reframing and rethinking distorted thoughts about situations and stressors, building positive expectations, and re-enforcing control over reaction to problems/situations, and strengthening the concept/belief in the ability of the mind and body to work together to create desirable changes in behavior/outcome. Clinical hypnosis allows the adolescent to gain a sense of control, increase self-esteem and competence, and reduce stress, therefore helping them to manage their physical and emotional well-being. For some problems, hypnosis may be the treatment of choice (e.g., enuresis, headaches, abdominal pain, procedural pain/anxiety, and adjustment reaction to stress). For more complex problems/conditions, hypnosis may be more adjunctive but a highly effective and important modality in the overall management.

## 6. Suggested Training

(1) American Society for Clinical Hypnosis. http://www.asch.net.
(2) National Pediatric Hypnosis Training Institute. http://www.nphti.org.
